# In Search of a Twenty-First Century Education Renaissance after a Global Pandemic

**DOI:** 10.1007/978-3-030-57039-2_1

**Published:** 2020-11-05

**Authors:** Fernando M. Reimers

**Affiliations:** Harvard Graduate School of Education, Harvard University, Cambridge, MA USA; Harvard Graduate School of Education, Cambridge, MA USA

## Abstract

The COVID-19 Pandemic renewed interest on the question of what goals should be pursued by schools in a world rapidly changing and uncertain. As education leaders developed strategies to continue to educate during the Pandemic, through alternative education arrangements necessitated by the closure of schools, the question of re-prioritizing curriculum became essential. In addition, the anticipated disruptions and impacts that the Pandemic would cause brought the question of what capacities matter to the fore. This chapter reviews the history of mass education and examines the role of the United Nations and other international organizations advocating for schools to educate the whole child and to cultivate the breath of skills essential to advance individual freedoms and social improvement. The chapter makes the case that the aspiration to cultivate a broad range of competencies is not only necessary to meet the growing demands of civic and economic participation, but also critical to close opportunity gaps.

The development of a science of implementation of system level reform to educate the whole child is fundamental to close the growing gap between more ambitious aspirations for schools and the learning opportunities that most children experience and that are at the root of their low levels of knowledge and skills as demonstrated in international comparative assessments.

Implementation strategies need to take into account the stage of institutional development of the education system, and align the components and sequence of the reform to the existing capacities and structures, while using the reform to help the system advance towards more complex forms of organization that enable it to achieve more ambitious goals. The chapter makes the case for examining the implementation of large scale reforms in countries at varied stages of educational development in order to overcome the limitations of the current knowledge base that relies excessively on the study of a narrow range of countries at similar levels of development, many of them with stagnant or declining performance of their students in international assessments of knowledge and skills.

Effective implementation requires also coherence across the various levels of governance of the education system and good communication and collaboration across a wide spectrum of stakeholders. Such communication can be facilitated by a good theory of mind of how others view reform. A reform can be viewed through five alternative frameworks: cultural, psychological, professional, institutional and political, or through a combination of those, and each reform is based on elements reflecting one or several of those frames. Understanding these frames, can help better understand how others view change, thus facilitating communication and the development of a shared theory of change.

The chapter concludes describing the methods of this study and introducing the six large scale reforms examined in the book.

## The Coronavirus Disease Pandemic and a New Consciousness About the Power of Education to Improve the World

On March 11, 2020 the World Health Organization declared the Coronavirus outbreak a pandemic, likely to spread to every country in the globe. At that time, 114 countries had reported that 118,000 people had contracted COVID-19, the illness caused by the SARS-CoV2 virus and that 4300 people had died (Branswell & Joseph, 2020). Over the subsequent months, the Pandemic would ravage the world, infecting people, causing deaths, stressing the capacity of hospitals to care for the sick, and significantly disrupting many domains of human activity, as a result of the social distancing measures imposed by public health authorities in an attempt to slow down the velocity of the transmission of the virus. These measures were deeply disruptive to education systems as, in country after country, schools closed down.

The closure of schools led many to express concerns about the potential learning loss and about the lack of sustained engagement with learning that would result, and about the fact that there would be significant differences among groups of children within countries and across countries in the amount of learning loss and in the level of school disengagement and potential dropout. This concern is noteworthy because it indicates that, around the world, schooling had become widely ingrained as an expectation of the normal experience of growing up. That amidst a global Pandemic in which lives were at stake, serious attention focused on what this would do to the opportunities of children and youth to study is significant. A similar concern with education as a priority would have been unlikely during the Influenza Pandemic of 1918–1919, a time when only 39% of the world population over the age of 15 had accessed some formal education, compared to 86% in 2020 (Roser & Ortiz-Ospina, 2013). The concern over education during COVID-19 demonstrates that the notion that all children should be schooled had become normalized, a significant global achievement.

To mitigate learning loss and learning disengagement, schools and education systems created alternative means to sustain opportunities for students to continue learning remotely. These involved using a variety of resources and technologies, from instructional packages, to radio and television, to online learning. In two survey studies I conducted, in partnership with Andreas Schleicher at the OECD, I found that between the end of March of 2020 and the end of April of 2020, many countries around the world had gone from not having a strategy of educational continuity, to having put in place strategies of educational continuity using alternative means of delivery (Reimers & Schleicher, 2020a, b). Notwithstanding the fact that these alternative delivery systems reached different children with various levels of success, and that many questions remain about their efficacy in supporting learning, these strategies demonstrated remarkable capacity of educators for rapid innovation. As educators developed these alternative means of delivery, the question of what should be taught, whether new priorities should be established in the curriculum to account for the diminished capacities of the alternative systems deployed, became critical. Should the strategies of continuity focus on ‘the basics’, perhaps the basic literacies of reading, math and science, or should they instead focus on emotional and social development, or some other combination of ‘breadth of skills’? The timeless question of ‘what is education for’ received renewed and considerable attention as schools and governments sought to sustain educational opportunity even as children could not attend school.

In addition, as many children were confined to their homes with their parents or guardians, the interruption of school attendance created a widely shared experience, for a vast number of people around the world, of observing up-close the process of engaging with school curriculum, as delivered by these quickly put together alternative means. In many cases, parents or guardians supported their children with their studies at home. This everyday experience with schooling for many, furthered interest on the question of what knowledge and skills students were gaining and, by extension, also interest on what skills they should be gaining, particularly as the alternative forms of continuity made evident that some children were better prepared than others to learn independently and remotely.

These questions about education purposes and the means to best achieve them are likely to stay with us not just during the Pandemic, but in the immediate post-Pandemic aftermath and beyond. Anticipating that the Pandemic will leave a number of lingering challenges, it is likely that even after the Pandemic is under control, a return to schooling will involve not just resuming formal education as it was left before the Pandemic, but to schools that will have to be reimagined to better address needs created by the Pandemic or made more evident by it, such as helping students develop the skills to learn independently, or addressing visible societal challenges such as poverty, social inequality, racism and bigotry, political polarization, national and international conflicts, or climate change.

Furthermore, the question of how to make schools more relevant will be intertwined with the question of how to develop institutional capacity and of resources, as it becomes apparent that the Pandemic will be followed by a period of financial austerity, in which societies will have to meet many needs with resources that are now further constrained by the slowdown in economic activity and by the costs of attending to the public health crisis. Schools will have to meet the new scrutiny and heightened expectations of how to better prepare students for a volatile and rapidly changing world in a context of clear resource and institutional constraints. The purpose of this book is to contribute to address these questions with some lessons drawn from the comparative analysis of several recent large scale education reforms that attempted to make schools more relevant to the needs of a changing world.

## How Should We Educate All Children?

The concern over educational continuity during the Pandemic made evident that there is today widespread global agreement that all children should be educated. That we have reached such consensus should not be underestimated, for such consensus was elusive not just during the Pandemic of 1918–1919, but even at the time the goal of educating all children was included as one of the thirty rights contained in the Universal Declaration of Human Rights, adopted at the United Nations in 1948. In the seven decades since its adoption few of the ideas reflected in the remaining twenty-nine articles have seen greater universal adoption.

The seismic shift represented by the universalization of the idea that all children should be educated, however, has not translated into the same agreement with respect to how to do this. To be sure, as the institution of schooling has emerged as the preferred mean to advance the right to education, schools have become ubiquitous and policies and programs have managed to include most children in school for a significant period of their lives. The universal consensus ends here, for the schooling experiences of children differ vastly for different children born into different circumstances in the same societies, and for children across the world. In part those differences result from lack of capacity of some schools to achieve their goals. For public schools, these differences reflect limited institutional capacity of governments to support all schools as necessary. But the differences are also by design, reflecting contention regarding what goals schools should advance, and what particular learning outcomes they should pursue for different students.

One such contention, arguably at the root of many others, concerns whether schools should provide students a fundamental instruction in the basic literacies of reading and writing, and numeracy, or whether they should endeavor to equip students with a broader set of capacities. The contention is aggravated by limitations in education funding and in the capacities of the public system. It is one thing to argue that all children should be broadly educated, but quite another to reach consensus on how to fund the necessary level of resources to be able to do this or to know how to organize schools and support teachers so that this aspiration translates into real learning experiences for students.

The question then of determining how to educate all children sits at the intersection of questions about goals, institutional capacity and resources. Facing the obvious challenge of resource constraints priorities are essential, anticipating greater complexity and costs in implementing reforms with ambitious goals, it may be tempting to argue that the basics should come first. From that line of thought one would argue that the first order of business for school systems should be to teach the foundational literacies and that more expansive goals should only be considered after succeeding at teaching the basics. An expression of that argument is found in the recent World Bank Development report documenting a ‘global learning crisis’ focusing on basic literacy and numeracy (World Bank, 2018). Aligned with this concept, the World Bank has introduced the concept of learning poverty, understood as the capacity to read a simple text by the age of 10. Given that 53% of all children in low and middle income countries are unable to read a simple text (World Bank, 2019), the urgency of addressing this target is self-evident. In practice, many governments and international development institutions prioritize the basic literacies. A recent report from the Global Partnership for Education, the largest partnership to support and fund education in the developing world, states that ‘the learning outcomes that are the focus of GPE 2020 [their current strategic plan] are de facto in relation to the foundational areas of literacy and numeracy’ (Global Partnership for Education, 2020, 1). Similar priorities are observed in countries such as the United States in which governments have pursued reform strategies that heighten accountability for schools to deliver on the basic literacies, an approach which has been shown to limit the breath of the curriculum, teach to the test, and lower assessment standards to show spurious improvement (Ravitch, 2010).

What the argument of prioritizing basic literacy or numeracy means in practice, in spite of its face value appeal, is that students attending the most endowed or better functioning schools, or school systems, have opportunities to develop a breath of skills which are denied children attending more precarious systems. This makes clear how the self-evident need to prioritize the basics because it is what is most ‘feasible’ quickly becomes an issue of ‘equity’. This is arguably the situation we are at present: some children have opportunities to develop a breath of skills that others lack. These inequities are found within countries as well as across countries. As the expanded range of skills that only some students develop translates into economic advantages, those educational inequalities in turn translate into socio-economic inequalities (Deming, 2017; Taylor et al., 2017).

Confirming such limitations of an approach to focus on the basics, a recent study of education reform in Massachusetts, widely considered a national leader in the standards based education reform movement in the United States, demonstrated that over the last 20 years, even as overall educational attainment had increased, the gaps between White and Latino students and between White and Black students and between Low and High income students in access to college and college completion had increased, with considerable impact in subsequent labor market earnings post college graduation, even for students who had comparable levels of performance in the Massachusetts Comprehensive Assessment System (MCAS).

Among students with the same performance in the MCAS, students from low-income families are significantly less likely to complete a 4-year college degree than their peers from high-income families (Papay et al., 2020 p. 22). The authors of the study offer several complementary explanations for these gaps, beginning with the different experiences of students in high school:

Low-income students are increasingly concentrated in schools in which most of their classmates also are living in poverty. Such schools tend to have fewer resources for college guidance and fewer students who plan to attend a 4-year college. These schools are also more likely to be at risk of sanctions because of low MCAS scores, which may lead educators to focus on increasing scores using strategies that do not promote mastery of critical academic and social skills important in college. (Papay et al., 2020, p. 23).

There are similar gaps associated with race. While Black students are 2% more likely to enroll in a 4 year college than their white peers, they are 11% less likely to graduate, and Hispanic students are 15% less likely to enroll in a 4 year college and 20% less likely to graduate than their white peers. (Papay et al., 2020, p. 24).

Given that focusing on ‘basic skills’ limits opportunities for students, it follows that in a world demanding breath of skills for full economic and civic participation, there is no choice but to provide all children the opportunity to develop an expanded range of skills, as anything else would amount to giving the most marginalized children access to the opportunity to gain skills that are increasingly irrelevant, of little value to advance oneself and one’s community while their more advantaged peers receive opportunities to gain skills that truly matter. Such view in favor of expanding the goals of education for all is reflected in the United Nations Development Goal for Education, goal 4, and in particular in target 4.7

4.7 by 2030 ensure all learners acquire knowledge and skills needed to promote sustainable development, including among others through education for sustainable development and sustainable lifestyles, human rights, gender equality, promotion of a culture of peace and non-violence, global citizenship, and appreciation of cultural diversity and of culture’s contribution to sustainable development (UN, 2020)

## The Need for a Science of Implementing Twenty-First Century Education and Deeper Learning Reforms

While the moral argument to provide all children the opportunity to develop a breath of skills is compelling, there is no easy way to resolve the conundrum of whether to align education systems to teach the basics or to teach more advanced skills, when the needs are many, the resources few and institutional capacity is limited. This conundrum is aggravated by limitations in our knowledge of how to translate this aspiration of teaching a broad range of skills in practice, for all students, at scale. This is the kind of dilemma the creation of alternative education systems to sustain educational opportunity during the Pandemic made painfully clear, some systems narrowed down curricular goals because lack of knowledge of how to achieve them with the precarious systems which had been rapidly developed during the emergency.

But for many children, the education systems they experience are always precarious. This is most often because of lack of resources of one sort or another, but also because of lack of knowledge of how to do better withing those constrains. This dilemma will continue in the coming years of austerity as we wrestle with the question of what should we teach all children and how. Examining how different countries approached the challenge of elevating the goals of education for all children at scale in recent years is one way to inform the policy debate on this topic. The purpose of this book is to explain what strategies where followed by six system level reforms to broaden curricular goals, and to examine their implementation.

The reforms studied in this book preceded the Covid-19 Pandemic, and there may be inherent limitations in that sense in extrapolating from even that recent past to a world that may be considerably changed as a result of the Pandemic. In some respect however these reforms wrestled with the dilemma of what does it mean to expand the goals of education in ways that anticipated the thorny challenges now confronted by most nations because of the new consciousness about the importance of intentionally leading educational change with clear goals for what students should learn and why caused by the Pandemic.

More than knowledge about what goals for education are pursued by various nations is needed to develop sound strategies of educational change to help all students gain the breadth of skills necessary in a rapidly changing world. What is direly needed is knowledge about how the strategies to advance more ambitious goals are implemented in practice. Even if the knowledge of what goals countries pursue is accompanied by knowledge about the levels of student achievement in assessments of those domains, there are limits to such type of comparative analysis because countries face different education needs and their education systems have differing resources and institutional capacity. For example, some countries still grapple with completing the provision of access to all students of school going age, some facing growing populations of children which create demands to recruit more teachers, while others benefit from demographic transitions, which allow them to concentrate resources on fewer children.

Because education systems are at different stages of institutional development, knowing what goals are pursued by high performing systems can be of limited value to inform efforts of improvement. An education leader cannot just wish that their education system looked like Singapore’s, one of the nations where students perform at high levels in international assessments of knowledge and skills in the areas of language, math and science. In order to develop clear action steps to make progress in educating all children in a particular system it is necessary to understand how the institutions of education develop over time, how developmental trajectories through which school staff, teachers and education leaders, and schools as organizations, lead to greater capacity to take on more ambitious goals. Ideas about those institutional developmental trajectories must define the details of implementation, including sequence and speed of change. Are there some elements in the development of the capacity of an education system which must be in place before others can be? How quickly can education systems progress towards greater capacity? While, arguably, a system needs a basic capacity to deliver the fundamentals of literacy and math education before it takes on more challenging education goals, is it the case that once they build that capacity that they will be better able to help students gain twenty-first century skills? We lack a sound theory of the development of education systems which can help answer those questions. We also need more knowledge about the details of implementing reforms to help students gain twenty-first century skills.

Since countries are at various stages of educational development, in terms of their education priorities and institutional capacity, understanding how systems at various stages of educational development implement strategies to serve the learning needs of students can contribute to theorize what kind of strategies are appropriate at various stages. This can help understand how systems can build the capacity of teachers and administrators to pursue ambitious goals, and how such efforts at capacity building are supported by other institutional reforms.

In spite of such absence of sound theories that can inform the development of strategies of system level transformation, governments around the world are embracing broader goals for the curriculum in many different nations. Predictably, the absence of an adequate theory to inform the development of strategies to achieve the adoption of more ambitious education goals, results in a gap between policy intent, implementation and practice. A recent study of national education mission statements in 113 countries, found that 86% of them included evidence of a commitment to the development of a broader range of skills with significantly less evidence of implementation of efforts to translate those goals into changed educational cultures in schools.

This suggests that countries are recognizing the importance of breadth of skills, at least in terms of aspirational statements reflected in policy documents. However, only a few countries are consistently identifying skills at both policy and practice levels. (Care, Kim, Anderson, & Gustafsson-Wright, 2017, p. 5).

The scan establishes conclusively the ubiquity of the breadth of skills movement through education systems. However, it is very much a work in progress. Many countries do not yet delineate how skills are expected to align with curriculum, nor do they include expectations for how these skills are to develop and mature, in the way they do for traditional subjects. (Ibid, p. 6).

Similar conclusions regarding the growing interest in twenty-first century skills and the challenges in implementation are presented in a landscape review conducted by the Global Partnership for Education, which works to support educational reform in countries with low levels of income per capita. The review notes that in a sample of education sector plans in 15 countries in Africa and Asia supported by the Partnership, all of them mention twenty-first century skills as education priorities, however the same review also notes that only three of those fifteen countries had implementation plans that included activities that could support the implementation of twenty-first century skills (Global Partnership for Education, 2020, vi).

In addition, while existing implementation may focus on some components (for example, teacher training), it appears that there is a lack of knowledge and experience of how to approach implementation at a whole sector or system level, including practical frameworks and guidance for doing so. (Ibid).

What appears to be absent in this ecosystem is work on integrating 21 century skills from a systemwide implementation lens. Despite the proliferation of initiatives across the partnership, there is little in the way of research, knowledge sharing, capacity development and advocacy around what it means to integrate and promote 21 century skills throughout an education system, particularly in developing countries. (Ibid).

Also reflecting this growing interest in broadening the curriculum, the International Association for the Evaluation of Educational Achievement, responsible for the oldest comparative cross-national surveys of student knowledge and skills, is planning a comparative, curriculum mapping study to examine opportunities to develop twenty-first century skills in the curriculum of participating countries.

Given this growing interest in broadening the goals of the curriculum, and the observed gap in implementation strategies to advance such goals, it is helpful to take stock of how governments implement reforms that broaden curriculum goals. What are the particular competencies they emphasize? What strategies do they adopt to support teachers and schools so they can help their students develop those competencies? Do countries that prioritize the basic literacies eventually transition to teaching twenty-first century skills, once they have succeeded in teaching the basics? How do they sequence those reforms? How do they pace them? What are the results in countries with low levels of institutional capacity and resources, when they prioritize twenty-first century skills?

Examining these questions is the purpose of this book, as we study reforms aimed at broadening curriculum goals in a group of diverse countries. We look at system level reforms in jurisdictions where students already achieve at high levels in international assessments of the basic literacies, such as Singapore and Ontario, Canada, as well as in nations where students achieve a much lower levels, such as Kenya, Mexico, Punjab-Pakistan and Zimbabwe. We examine system level reforms which focus on strengthening the capacity to teach the basics, as in Ontario and Punjab, as well as reforms that aim at building the capacity to teach a much broader set of competencies and skills, such as Kenya, Mexico, Singapore and Zimbabwe. We look at systems at very different levels of spending per student, and at reforms at various points in the cycle of policy implementation, some just starting as in Kenya and Zimbabwe, others such as Mexico’s struggling to survive a governmental transition, and others such as Ontario, Punjab and Singapore that have been in place for an extended period of time. From the comparative study of these reforms we draw lessons on the implementation of reforms to teach twenty-first century skills at scale in diverse settings.

The choice of these countries is to some extent arbitrary. The studies of the reforms presented in this book originated in a graduate course I teach on comparative education policy analysis at Harvard University. The course draws a very diverse group of students, many of them in the International Education Policy Program I direct at the Harvard Graduate School of Education. Students are naturally drawn to carry out studies in countries in which they have experience or contacts to access the kind of information that this study required. Through this somewhat serendipitous approach, we ended with a collection of countries that are diverse in various useful ways, in this way expanding the knowledge drawn from existing studies of large system change which focus on countries all at similar levels of economic and institutional development, of which a preponderance are based on a narrow set of high income countries.

In examining these various reforms, we draw on a theoretical framework that sees the process of educational change as encompassing five perspectives, five ways of thinking about reform: cultural, psychological, professional, institutional and political (Reimers, 2020c). This comparative study of six large scale reforms examines which of these perspectives are reflected in the strategies pursued by governments as they seek to strengthen the capacity of the public education system to teach ambitious goals.

## The Global Education Movement and the Right to Education in a Changing World

The current consensus on the universal right to education is squarely a result of the international architecture to promote development created at the end of World War II. The same international institutions have played a pivotal role in shaping ideas about how students should be educated and to what ends. The inclusion of education as a right in the Universal Declaration adopted at the third session of the United Nations General Assembly on December 10, 1948 spearheaded a global movement to educate all children (Reimers, 2017). The United Nations Education, Culture and Science Organization (UNESCO), the specialized agency established to support the advancement of that right, has played a pivotal role leading this global education movement through the strategic exercise of five functions:

- **Generating and disseminating ideas** – anticipating and responding to emerging trends and needs in education, and developing education policies based on research and country priorities,
- **Developing and promoting the adoption of education standards** – developing policies and practices,
- **Serving as a clearinghouse** – promoting the development, implementation and dissemination of successful educational policies and practices setting norms and standards and providing support in their implementation,
- **Building capacity** – providing technical co-operation to develop the capacity of member states to achieve their national education goals,
- **Catalyzing international co-operation** – initiating and promoting dialogue and exchange among education leaders and stakeholders.

Those actions by UNESCO, as representative and steward of the global commitment to the right of education, and of national and local governments, organizations of civil society and other international organizations, steered a global education movement which transformed the experience of humanity, creating an institution, the school, designed to provide children and youth opportunities to learn and norming that all children would spend a significant period early in their lives in that institutional creation. The concern over the education of children during the COVID-19 Pandemic demonstrated the success of that global education movement in normalizing the idea that education was a human right.

As a result of this global education movement, global access to education increased dramatically, especially in the developing world. In the 1950s and 1960s UNESCO convened meetings of Ministers of Education, and of Finance, to advocate for the universalization of basic education. This advocacy, and the adoption of global norms and resolutions incorporating that right, resulted in legal and regulatory reforms in many countries enshrining the right of education. UNESCO then promoted the adoption of specific standards, stipulating for example the duration of compulsory education or creating the International Standard Classification of Education, a framework to organize information on access at different levels and modalities of education. UNESCO also monitors country’s enrollment rates and disseminates such information, as a way to further reinforce country’s commitments to implement programs to achieve the agreed upon resolutions. In its role as a clearinghouse of ideas and good practices, UNESCO documents practices which have contributed to the achievement of the goal of universalizing access or closing equity gaps, for example the creation of double shift schools, or cluster schools to rapidly expand access through better utilization of existing infrastructure. Through a variety of courses and training programs it developed the capacity of government staff who could help design and implement policies and programs that contributed to the achievement of the universalization of the right to education. Finally, UNESCO mobilized other international agencies to support countries in the achievement of those goals.

**Fig. 1.1:**
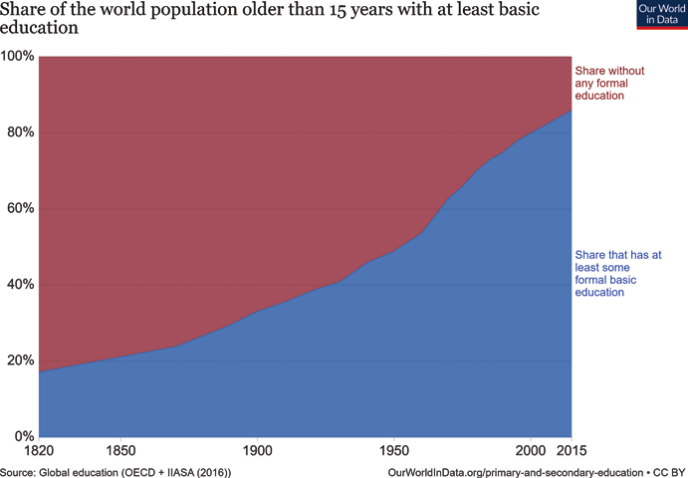
Share of the world population older than 15 years with at least basic education. (Source: Roser, M. and E. Ortiz-Ospina (2013). “Primary and Secondary Education” Published online at OurWorldInData.org Retrieved from https://ourworldindata.org/primary-and-secondary-education)

The impact of this global education movement is nothing short of remarkable. I have elsewhere defined it as the most significant silent revolution experienced by humanity (Reimers, 2017). Whereas prior to the creation of UNESCO less than half of all children had the opportunity to attend school, seven decades later, most of them had the same opportunity as shown in Fig. 1.1.

Figure 1.1 shows the percentage of world the population over 15 years of age which had accessed at least some formal education, increasing from under 20% in 1820 to 85% in 2015. The figure shows that most of the expansion in access to school took place during the 1950s and 1960s. Note that those increases in relative access took place even as population grew considerably. The most dramatic increases, in population as well as in access, were in the developing world, where levels of access to school were lower. The result was a significant increase in the average level of educational attainment of the population. As the global education movement brought to school children whose families had not traditionally been schooled, and as the demands for meaningful participation in society increased, new questions emerged about what should be the purpose of educating all children.

With respect to this question of purpose, UNESCO played also an important guiding role. The foundational answer to the question of why should all children be educated is provided in the text of the Universal Declaration of Human Rights itself, in article 26 which spells out the right to education and which directs the expansion of education to the full development of the human personality and to promoting understanding, tolerance and friendship among nations, racial or religious groups, as well as to furthering the activities of the United Nations to maintain peace:

**Article 26.**

1. Everyone has the right to education. Education shall be free, at least in the elementary and fundamental stages. Elementary education shall be compulsory. Technical and professional education shall be made generally available and higher education shall be equally accessible to all on the basis of merit.
2. Education shall be directed to the full development of the human personality and to the strengthening of respect for human rights and fundamental freedoms. It shall promote understanding, tolerance and friendship among all nations, racial or religious groups, and shall further the activities of the United Nations for the maintenance of peace.
3. Parents have a prior right to choose the kind of education that shall be given to their children. (United Nations, 1948)

This emphasis on educating all in order to advance human rights, tolerance and peace has been an ongoing concern of UNESCO, reaffirmed at the 1974 General Conference in the “Recommendation concerning Education for International Understanding, Co-operation and Peace and Education relating to Human Rights and Fundamental Freedoms”. The recommendation proposed the following guidelines for education policy:

- An international dimension and a global perspective in education at all levels and in all its forms;
- Understanding and respect for all peoples, their cultures, civilizations, values and ways of life, including domestic ethnic cultures and cultures of other nations;
- Awareness of the increasing global interdependence between peoples and nations;
- Abilities to communicate with others;
- Awareness not only of the rights but also of the duties incumbent upon individuals, social groups and nations towards each other;
- Understanding – of the necessity for international solidarity and cooperation;
- Readiness on the part of the individual to participate in solving the problems of his community, his country and the world at large.

5. Combining learning, training, information and action, international education should further the appropriate intellectual and emotional development of the individual. It should develop a sense of social responsibility and of solidarity with less privileged groups and should lead to observance of the principles of equality in everyday conduct. It should also help to develop qualities, aptitudes and abilities which enable the individual to acquire a critical understanding of problems at the national and the international level; to understand and explain facts, opinions and ideas; to work in a group; to accept and participate in free discussions; to observe the elementary rules of procedure applicable to any discussion; and to base value judgments and decisions on a rational analysis of relevant facts and factors.

6. Education should stress the inadmissibility of recourse to war for purposes of expansion, aggression and domination, or to the use of force and violence for purposes of repression, and should bring every person to understand and assume his or her responsibilities for the maintenance of peace. It should contribute to international understanding and strengthening of world peace and to the activities in the struggle against colonialism and neo-colonialism in all their forms and manifestations, and against all forms and varieties of racialism, fascism, and apartheid as well as other ideologies which breed national and racial hatred and which are contrary to the purposes of this recommendation. (UNESCO, 1974).

To advance ideas about the purpose of education for all, throughout its history, UNESCO has thrice established commissions tasked with producing guiding frameworks. The first commission established at UNESCO’s General Conference in 1970 and chaired by former minister of education of France Edgar Faure, produced a report titled ‘Learning to Be’ which made the case for lifelong learning as a way to contribute to the full development of people as capable of agency to direct their own lives, promoters of democracy and world citizens (Faure et al., 1972, 158). The second commission, established at the 1991 General Conference, was chaired by Jacques Delors, a former President of the European Commission, proposed that education should be organized around four goals: learning to know; learning to do; learning to live together; and learning to be, of which the report assigned the greatest importance to learning to live together (Delors, 1996). In 2019, UNESCO established another commission to produce a report on the goals of education, The Education on the Futures of Education Commission, chaired by the President of the Federal Democratic Republic of Ethiopia Sahle-Work Zewde. The Faure and Delors reports reflect a humanist vision of education, very much in line with the values of the Enlightenment that emphasize the equal rights of all people, the capacity of people to become architects of their lives, and their ability to rule themselves and improve the world.

The ideas contained in the Delors report, that the purposes for school learning should be broadened to prepare students for a changing and uncertain world, found resonance throughout the world. While the Delors report was not discussed by the Executive Board of UNESCO, it was translated into about 30 languages, and generated initiatives in 50 countries, stimulating the development of indicators of lifelong learning, and position papers on education, as well as pilot projects, and the four pillars of learning became a catchphrase in policy documents (Elfert, 2015, 94).

Alongside this work of UNESCO, since the early 1990 a number of international organizations, governments and other institutions developed frameworks and advocated to broaden the range of skills that schools should cultivate. These reflected several strands of ideas: a growing interest in the socio-emotional development of learners and in the development of life skills, an interest in more active forms of civic engagement, and an interest in the development of skills that allowed economic participation as workplaces were transformed by technology.

In 1994, a group of educators in the United States, formed a consortium to establish high quality evidence on ways to support socio emotional learning in schools, the Collaborative for Academic, Social, and Emotional Learning (CASEL). In 1997, CASEL partnered with the Association for Supervision and Curriculum Development, a large professional association of educators in the United States, producing guidelines for educators on how to promote socio-emotional learning in schools (CASEL, 2020). CASEL defines five core socio-emotional competencies: Self-awareness, Self-management, Social-awareness, Relationship skills and Responsible decision making.

Self-awareness consists of “the ability to recognize one’s own emotions, thoughts, and values and how they influence behavior. The ability to accurately assess one’s strengths and limitations, with a well-grounded sense of confidence, optimism, and a ‘growth mindset’” (CASEL, 2020)

Self-management is “The ability to successfully regulate one’s emotions, thoughts, and behaviors in different situations – effectively managing stress, controlling impulses, and motivating oneself. The ability to set and work toward personal and academic goals.” (Ibid.)

Social-awareness is “The ability to take the perspective of and empathize with others, including those from diverse backgrounds and cultures. The ability to understand social and ethical norms for behavior and to recognize family, school, and community resources and supports.” (Ibid.)

Relationship skills comprise “The ability to establish and maintain healthy and rewarding relationships with diverse individuals and groups. The ability to communicate clearly, listen well, cooperate with others, resist inappropriate social pressure, negotiate conflict constructively, and seek and offer help when needed.” (Ibid).

Responsible decision making involves “The ability to make constructive choices about personal behavior and social interactions based on ethical standards, safety concerns, and social norms. The realistic evaluation of consequences of various actions, and a consideration of the well-being of oneself and others.” (Ibid).

Similar socio-emotional goals for schools were advocated in 1999 by the World Health Organization in an inter-agency report underscoring the importance of promoting psycho-social or life skills to help students deal with the demands and challenges of life, in particular to empower children and youth at risk to protect their rights. The report identified five basic areas of life skills: decision making and problem solving, creative and critical thinking, communication and interpersonal skills, self-awareness and empathy and copying with emotions and stress (WHO, 1999, 1).

Also in 1999, UNICEF developed a framework for ‘child friendly’ schools and educational systems, based on the Convention on the Rights of the Child. The framework reflects a student centered, whole-child vision of education, aligned with a broad set of educational competencies (UNICEF, 2020). Building on UNESCO’s Delors Report, UNICEF has also developed a framework of life skills and citizenship to support the development of children in the Middle East that reflects an ambitious set of twelve core life skills aligned to the four pillars in UNESCO’s report. Learning to know, for instance, is reflected in Skills for Learning (creativity, critical thinking, problem-solving), learning to do in Skills for Employability (cooperation, negotiation, decision-making), learning to be in Skills for Personal Empowerment (self-management, resilience, communication) and learning to live together in Skills for Active Citizenship (respect for diversity, empathy, participation) (UNICEF, 2017, 4).

The Organization for Economic Cooperation and Development also contributed to the global dialogue on broader goals for education through the project of Definition and Selection of Competencies in the 1990s and the associated Programme of International Student Assessment launched in 1997, and more recently through the OECD 2030 Learning Framework, outlining an expanded set of competencies that could contribute to individual and collective wellbeing (OECD, 2020). This framework consists of a Learning Compass which includes “Core knowledge, skills, attitudes and values for 2030 will cover not only literacy and numeracy, but also data and digital literacy, physical and mental health, and social and emotional skills.” (OECD, 2020), building on those foundations are Competencies, which include knowledge, skills, attitudes and values that allow a person to act in coherent and responsible ways that change the future for the better (Ibid). Finally, transformative competencies allow students to develop and reflect on their own perspective and support learning and the capacity to take responsibility to create a better world.

The National Research Council of the United States, commissioned an expert panel to produce a scientific consensus report on skills variously termed ‘deeper learning’, ‘21st century skills’, ‘college and career readiness’, ‘next generation learning’, ‘new basic skills’ and ‘higher order thinking’ (National Research Council, 2012). The report, published in 2012, classified these skills as:

1. **Cognitive Skills**
  1.1. Processing and cognitive strategies
    Critical Thinking
    Problem Solving
    Analysis
    Logical Reasoning
    Interpretation
    Decision Making
    Executive Functioning
  1.2. Knowledge
    Literacy and communication skills
    Active listening skills
    Knowledge of the disciplines
    Ability to use evidence and assess biases in information
    Digital Literacy
  1.3. Creativity
    Creativity
    Innovation
2. **Interpersonal skills**
  2.1. Collaborative group skills
    Communication
    Collaboration
    Team Work
    Cooperation
    Coordination
    Empathy, Perspective Taking
    Trust
    Service Orientation
    Conflict Resolution
    Negotiation

2.2. Leadership
  Leadership
  Responsibility
  Assertive Communication
  Self-Presentation
  Social Influence

3. **Intra-personal skills**
  3.1. Intellectual Openness
    Flexibility
    Adaptability
    Artistic and Cultural Appreciation
    Personal and Social Responsibility
    Intercultural competency
    Appreciation for diversity
    Adaptability
    Capacity for lifelong learning
    Intellectual interest and curiosity
  3.2. Work Ethic/Responsibility
    Initiative
    Self-direction
    Responsibility
    Perseverance
    Productivity
    Persistence
    Self-Regulation
    Meta-cognitive skills, anticipate future, reflexive skills
    Professionalism
    Ethics
    Integrity
    Citizenship
    Work Orientation
  3.3. Self-efficacy
    Self-regulation (self-monitoring and self-assessment)
    Physical and mental health

Additional impetus for the expansion of education goals was provided by the compact of development adopted at the annual general conference of the United Nations, in September of 2015, at which participating governments embraced the goal of sustainable development, identifying seventeen goals and a series of specific targets, and highlighting the pivotal role education should play in the achievement of all other goals. The fourth Sustainable Development Goal ‘Ensure inclusive and equitable quality education and promote lifelong learning opportunities for all’, includes a target that explicitly focuses on education about sustainable lifestyles, mentioned earlier in this chapter.

More recently, in 2020, the World Economic Forum produced a brief outlining eight essential skills for the fourth industrial revolution:

1. Global citizenship skills: Include content that focuses on building awareness about the wider world, sustainability and playing an active role in the global community.
2. Innovation and creativity skills: Include content that fosters skills required for innovation, including complex problem-solving, analytical thinking, creativity and systems analysis.
3. Technology skills: Include content that is based on developing digital skills, including programming, digital responsibility and the use of technology.
4. Interpersonal skills: Include content that focuses on interpersonal emotional intelligence, including empathy, cooperation, negotiation, leadership and social awareness.
5. Personalized and self-paced learning: Move from a system where learning is standardized, to one based on the diverse individual needs of each learner, and flexible enough to enable each learner to progress at their own pace.
6. Accessible and inclusive learning: Move from a system where learning is confined to those with access to school buildings to one in which everyone has access to learning and is therefore inclusive.
7. Problem-based and collaborative learning: Move from process-based to project- and problem-based content delivery, requiring peer collaboration and more closely mirroring the future of work.
8. Lifelong and student-driven learning: Move from a system where learning and skilling decrease over one’s lifespan to one where everyone continuously improves on existing skills and acquires new ones based on their individual needs. (World Economic Forum, 2020, 4).

The advocacy of the various organizations involved in producing these diverse frameworks gradually caused governments around the world to revise and expand national standards and curriculum. A study of how curriculum goals had changed in the twenty first century in Chile, China, India, Mexico, Singapore and the United States found that in all these countries the curriculum had expanded to include a broader focus on cognitive, social and emotional competencies (Reimers & Chung, 2016). The same was found in a study of education reforms in Brazil, Finland, Japan, Mexico, Peru, Poland, Portugal and Russia (Reimers, 2020b).

UNESCO carries out periodic consultations to member states to assess the extent to which the goals of the 1974 recommendation are reflected in education policies and in the curriculum. The most recent consultation, to which 83 out of 195 member states responded, reports improvements in implementing the guiding principles of the 1974 recommendation. Among the respondents, 68% indicate that these principles are fully integrated in education policies, and an additional 51% indicate that they are somewhat reflected. All countries report that the curriculum includes goals reflecting peace and non-violence, 99% include human rights and fundamental freedoms, 96% include cultural diversity and 99% include environmental sustainability goals (UNESCO, 2018, figure 6). The same survey shows that there is a disconnect between the inclusion of these goals in the curriculum and the extent to which they are also incorporated in teacher education programs. Only 19% of the countries report that these goals are fully integrated in teacher preparation programs, and an additional 93% indicate that they are only somewhat integrated (UNESCO, 2018, figure 13).

An in-depth analysis of policy documents in ten countries with an expressed commitment to Education for Sustainable Development and Global Citizenship Education undertaken by UNESCO, revealed that in all these countries there are abundant references to both of these concepts, and that they are expressed in terms of cognitive, socio-emotional and behavioral dimensions (UNESCO, 2019). In the documents examined in these countries –Costa Rica, Japan, Kenya, Lebanon, Mexico, Morocco, Portugal, Republic of Korea, Rwanda and Sweden – there were almost twice as many references to Global Citizenship Education (representing about 60% of the references) than to Education for Sustainable Development (representing about 30%) across national laws, strategic plans and policies, national curriculum frameworks, programmatic documents and subject specific curriculum. These references were present across various subjects in the curriculum, and the emphasis on cognitive dimensions, relative to socio-emotional and behavioral domains, increased in secondary education (Ibid).

## Broader Curriculum Goals Don’t Teach Themselves. The Need for Effective Implementation Strategies That Augment Teacher Capacities

As the goals of the curriculum expand to encompass a broader range of skills and capacities, teacher capacity is increasingly recognized as the lynchpin to the success of these efforts to better prepare students for a world in which they will face greater cognitive and skills demands. This is the reason teacher professional preparation has become a crucial policy priority for many nations (Reimers, 2020a; Reimers & Chung, 2016). While there is evidence that teacher professional development can help teachers develop the pedagogical skills to educate the whole child (Reimers & Chung, 2018) and while many teachers receive professional development, it is clear that many teachers lack the capacities to enact the type of pedagogies which are known to cultivate some of the expanded skills considered essential to participate, civically and economically, in the twenty first century. As a result, many students lack opportunities for ‘deeper learning’ and the opportunities to develop the breadth of skills intended in the curriculum.

Cross national studies show that many students are poorly supported to develop cognitive or socio-emotional skills. In the last PISA study, less than 10% of the students can distinguish facts from opinions (OECD, 2019b, 3). In terms of their literacy skills, the assessment defines a threshold at which ‘students can identify the main idea in a piece of text of moderate length. They can understand relationships or construe meaning within a limited part of the text when the information is not prominent by producing basic inferences, and/or when the information is in the presence of some distracting information. They can select and access a page in a set based on explicit though sometimes complex prompts, and locate one or more pieces of information based on multiple, partly implicit, criteria. Readers at level 2 can, when explicitly cued, reflect on the overall purpose, or on the purpose of specific details, in texts of moderate length. They can reflect on simple visual or typographical features. They can compare claims and evaluate the reasons supporting them based on short, explicit statements.” (OECD, 2019b, 91). On average, 77% of the students in the OECD can read at this level or above, although there is much variation across countries in the percentage of students reading at various levels. In the Chinese provinces who participated in the study (Beijing, Shanghai, Jiangsu and Zhejiang), close to 95% of the students exceed this literacy threshold, that figure is above 80% in Australia, Denmark, Japan, Korea, New Zealand, Norway, Slovenia, Sweden, Taipei, the United Kingdom and the United States, and above 85% in Canada, Finland, Hong Kong and Poland. In contrast, more than 25% of the students were unable to read at this level in Chile, Colombia, Greece, Hungary, Iceland, Israel, Luxembourg, Mexico, the Slovak Republic and Turkey (OECD, 2019a, 92).

In Mathematics, the PISA study defines proficiency at level two or above as ‘students begin to demonstrate the ability and initiative to use mathematics in simple real-life situations… the ‘minimum level of proficiency’ that all children should acquire by the end of secondary education’ (OECD, 2019a, 105). While over 90% of students in Beijing, Shanghai, Jiangsu and Zhejiang, Hong Kong, Macao and Singapore and close to 90% in Estonia achieved at this level, in 21 countries only between 20% and 50% of the students did so (Ibid).

In Science, students below level 2 are unable to ‘draw on everyday content knowledge and basic procedural knowledge to identify an appropriate scientific explanation, interpret data, and identify the question being addressed in a simple experimental design’. On average in the OECD 78% of the students demonstrated this basic level of scientific knowledge or higher (OECD, 2019a, 115).

The pedagogical shortcomings of teachers to support their students disproportionally affect poor students. Socioeconomically disadvantaged students are more likely to perform at low levels than their more advantaged peers. For example, whereas on average among OECD countries, one in five students achieved below the threshold level for literacy, this figure was 36% for the poorest 25% of the students but only 11% for the wealthier 25% of the students. Socioeconomic background is significantly associated with student learning outcomes in all countries participating in PISA, with the sole exception of the province of Macao in China (OECD, 2019c, 54).

In spite of the relationship between socioeconomic background and learning outcomes, some disadvantaged students achieve at high levels in PISA, one in ten disadvantaged students achieves in the top 25% of the reading assessment. Those students have supportive parents, enthusiastic teachers, greater sense of self-efficacy and are in schools with a positive disciplinary climate (OECD, 2019c, 66). In half of the countries participating in the study, those students from low socioeconomic backgrounds who achieved at high levels were more likely to feel that they belonged in school (Ibid). These findings underscore the interdependence of various aspects of the educational experience, no one learns very much in a school where they don’t feel they belong, and students are more likely to apply themselves to their studies if they have more efficacy, or when they have a growth mindset, and the results of such effort will in turn reinforce their sense of efficacy and growth mindset. Effective teachers are able to educate the whole child, to support the development of their students in the cognitive, emotional and cognitive domains, and to do this for all their students.

These student learning outcomes are reflective of the opportunities they have to learn. Most teachers are better prepared to transmit content than to design and support the learning challenges which develop both higher order cognitive skills as well as socio-emotional skills. A recent study on teacher practices around the world conducted by the OECD, identifies that whereas most teachers report effectively deploying teacher centered strategies such as presenting a summary of recently learned content at the beginning of a lesson, setting goals at the beginning of instruction, explaining what they expected students to learn, or explaining how old and new topics are related, considerable fewer report using student centered pedagogies which engage students with tasks of high cognitive complexity, such as presenting tasks for which there is no obvious solution, giving tasks which require students to think critically, having students work in small groups to solve a problem or task, asking students to design their own procedures to solve problems, or giving students problems which require at least a week to complete. (OECD, 2019a).

These shortcomings in teacher’s pedagogical skills are confirmed by a recent survey to students, administered as part of the Programme for International Student Assessment. As a result of the varying levels of teacher skills to educate the whole child, the experience of school can be vastly different for different students. For instance, on average in the OECD 38% of students are in schools where at least 25% of the students are bullied at least a few times a month (OECD, 2019c, Table III.B1.2.3). The climate students experience in their classrooms varies also as a result of different teacher competencies, for instance on average across countries 29% of the students report that they can’t listen to the teacher in most or all lessons, 30% report noise and disorder in most or all lessons, 26% say the teachers have to wait a long time for students to quiet down in most or all lessons (Ibid, Table III.B1.3.3). Only 22% of the students strongly agree with the statement that the teachers enjoyed teaching the lesson, and an additional 50% agreed with the same statement. Only 15% of the students strongly agree with the statement that the enthusiasm of the teachers inspired them, and an additional 40% agree with the same statement. Only 24% of the students strongly agree with the statement that their teachers enjoy teaching the topics they were teaching, and an additional 55% agree with that statement. Only 25% of the students strongly agreed with the statement that teachers demonstrated enjoyment teaching, and an additional 49% agreed with the statement (Ibid, Table III.B1.5.3). These reports suggest that at least 25% of the students are in classrooms they find unsupportive, as confirmed by the fact that 75% of the students report that their teachers provide extra help when students need it (Ibid, Table III.B1.6.4).

These various experiences with teachers lead to various experiences of inclusion in school. Twenty percent of the students in the OECD agree or strongly agree with the statement that they feel like an outsider or left out of things at school; 29% disagree or strongly disagree with the statement that they feel like they belong in school; 20% agree or strongly agree with the statement that they feel awkward and out of place at school (Ibid, Table III.B.1.9.3).

While the student’s skills and experiences are indicative of shortcomings of many of their teachers, teachers themselves report a need for more effective professional development to effectively address a number of their professional challenges. For example, as a result of growing mobility and access, classrooms today are increasingly diverse, as shown in the following table displaying the percentage of teachers who teach in classes where more than 10% of the students come from homes where a different language to the language of instruction is spoken. In spite of such diversity, however, only a fraction of the teachers were prepared to teach in multicultural settings in their initial education, or feel well prepared to do so, or received professional development focused on teaching in multicultural settings. On average, for the OECD, 18% of the teachers are in classes with more than 10% of students who are learning in a second language; 35% of the teachers received preparation to teach multicultural classes in their initial preparation; 26% feel well or very well prepared to teach in a multicultural classroom; 22% received professional development addressing this are; 15% feel a high need for professional development in this area; and only 67% feel they can cope with the challenges of teaching a multicultural classroom (Table 1.1).

**Table 1.1:**
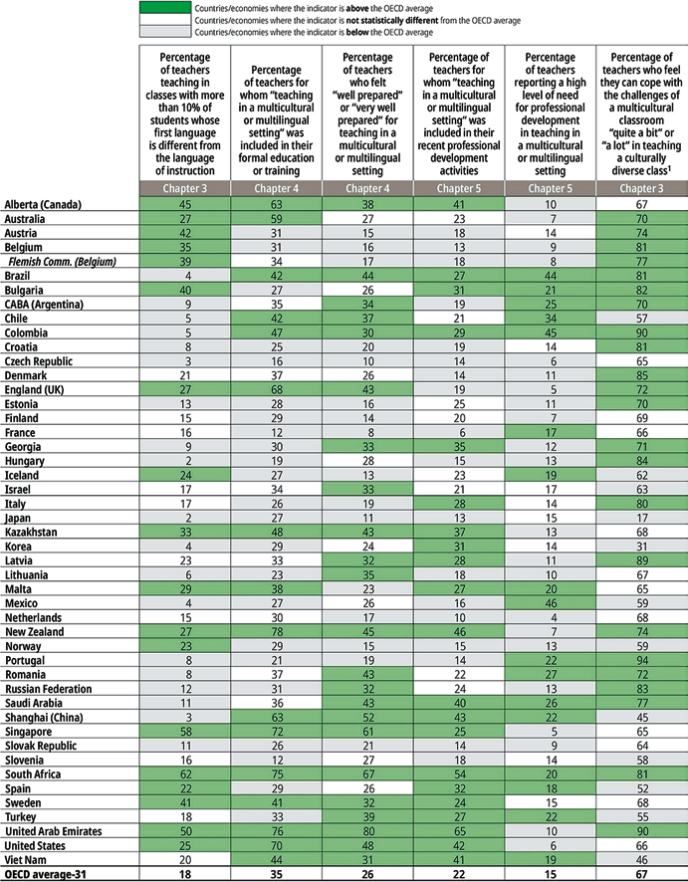
Teacher preparation to teach in multicultural settings

1. The sample is restricted to teachers reporting that they have already taught a classroom with students from different cultures.

Source: OECD, 2019a Figure I.1.2.

Similar needs for professional development are observed in teacher responses with respect to using information and communication technologies. On average, for the OECD, only 56% of the teachers indicate that the use of ICT was included in their initial preparation; only 43% feel well prepared or very well prepared to use ICT; only 60% indicate that the use of ICT was included in their recent professional development; 18% express a high need for professional development in using ICT for teaching; only 53% allow students to regularly use ICT for projects, and 25% of principals report shortage of digital technology for instruction. (OECD, 2019a, Figure I.1.1.)

Similar needs are evident with respect to teaching students with special needs. On average, for the OECD, 27% of the teachers teach in classrooms where more than 10% of the students have special needs; 67% have received preparation to teach in integrated classrooms as part of their professional development; only 44% feel well prepared or very well prepared to teach in inclusive classrooms; only 43% have received preparation to teach in inclusive classrooms in their recent professional development; 22% report a high level of need for professional development to teach in integrated classrooms and 32% of school principals report a shortage of teachers with competency to teach in integrated environments. Table 1.2 presents these results for all countries participating in the survey.

**Table 1.2:**
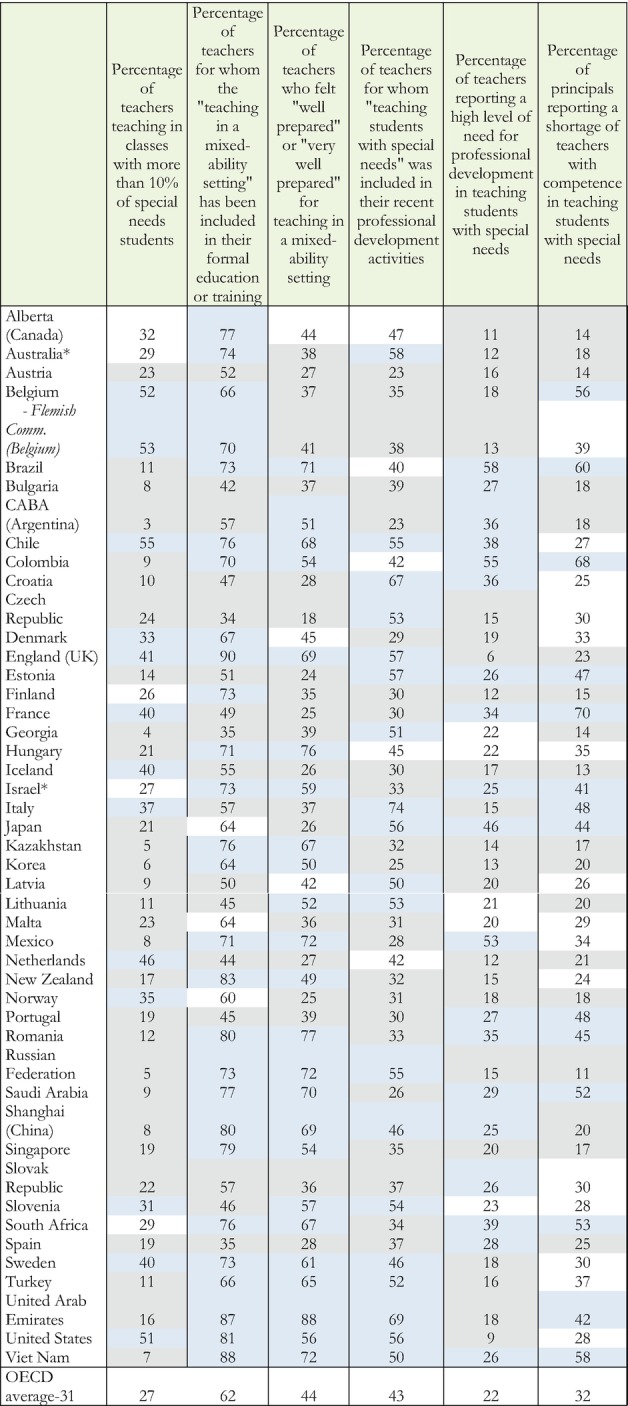
Teacher preparation to teach in inclusive classrooms

Source: OECD, 2019a, Figure I.1.3

Even with respect to simple instructional tasks, such as classroom management, many teachers feel inadequately prepared. On average for the OECD, only 72% of the teachers report learning about classroom management in their initial teacher preparation, and only 53% feel well or very well prepared to manage their classrooms; only 50% report that classroom management was addressed in their recent professional development; only 14% of teachers report a high need of professional development in their classrooms; 85% feel they can control disruptive behavior in their classrooms, but 29% report that they lose a lot of time because of student disruptions. Table 1.3 has detailed percentages of teachers who report adequate preparation to manage their classrooms in the countries participating in the study.

**Table 1.3:**
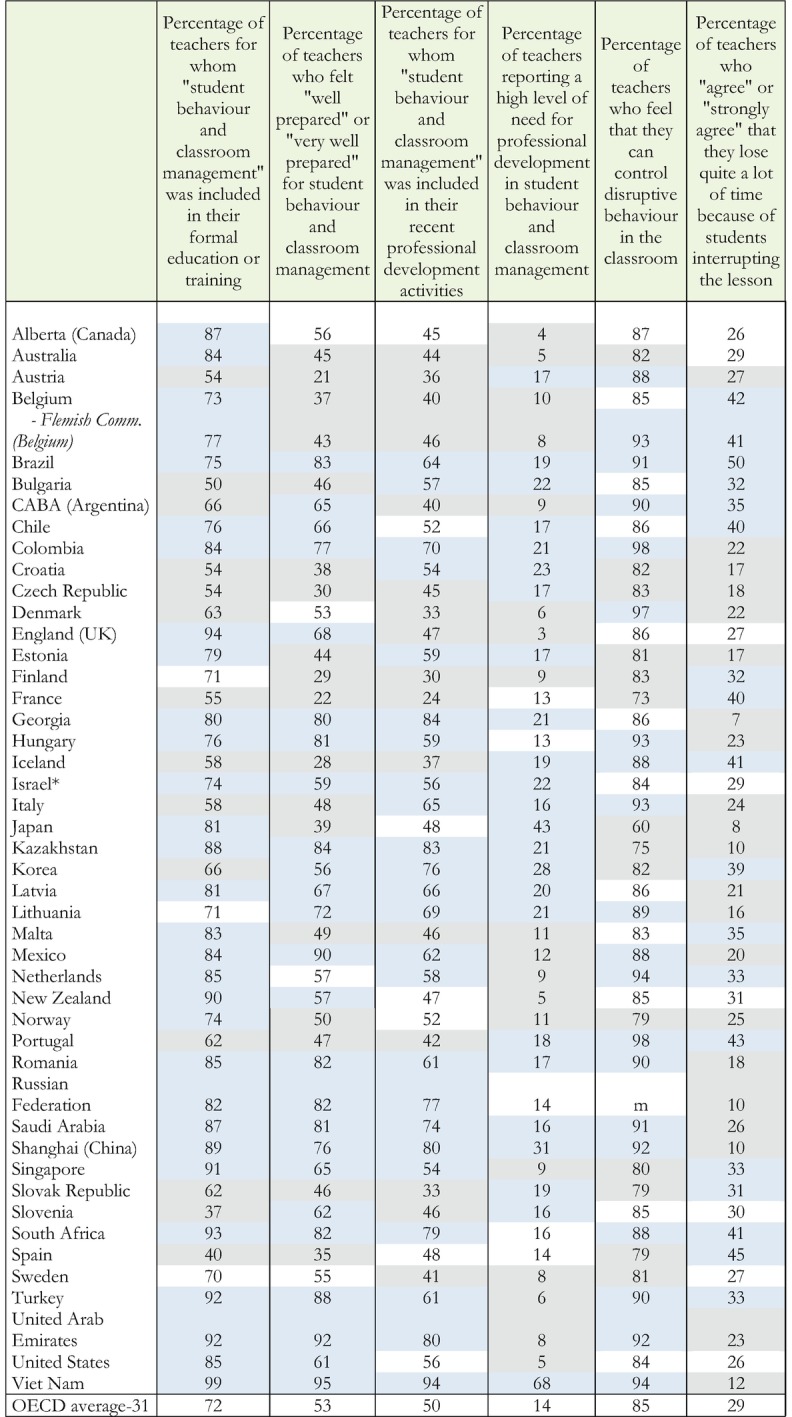
Teachers preparation to manage classroom discipline

Source: OECD, 2019a, Figure I.1.4

## The Limitations of What We Know to Develop More Effective Teacher Capacities to Educate the Whole Child

The fact that teacher education and development are recognized as important but many efforts to support teachers in learning new skills are insufficient to prepare them to meet the broader demands of more ambitious curricula is paradoxical, particularly given that much research has been conducted on teacher professional development and on the process of school improvement. Why is that research insufficient to guide more effective programs of teacher preparation?

**Fig. 1.2:**
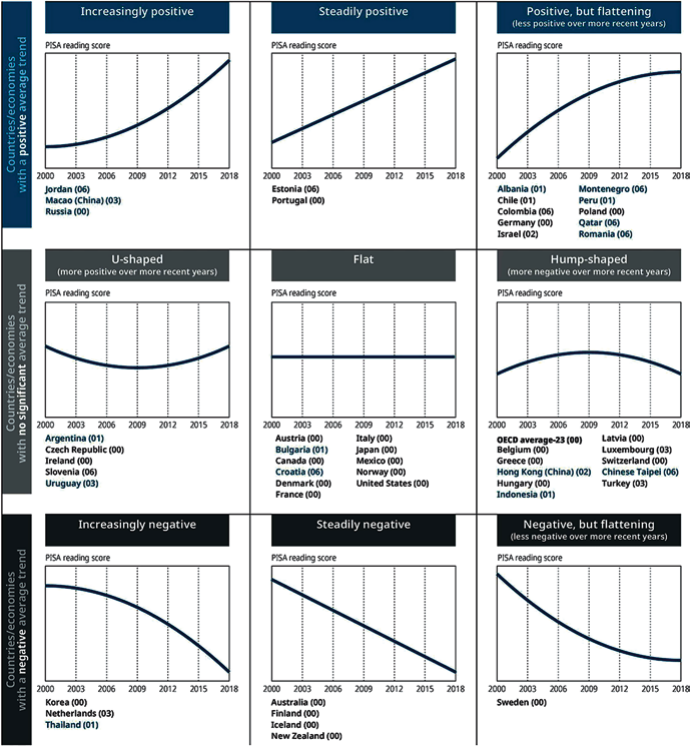
PISA results. (Source: OECD, 2019a Figure I.9.1) Notes: Figures are for illustrative purposes only. Countries and economies are grouped according to the overall direction of their trend (the sign and significance of the average three-year trend) and to the rate of change in the direction of their trend (the sign and significance of the curvature in the estimate of quadratic trends) (see Annex A7). Only countries and economies with data from at least five PISA reading assessments are included. Not all countries and economies can compare their students’ performance over the same period. For each country/economy, the base year, starting from which reading results can be compared, is indicated in parentheses next to the country’s/economy’s name (“00” = 2000, “01” = 2001, etc.). Both the overall direction and the change in the direction may be affected by the period considered. OECD average-23 refers to the average of all OECD countries with valid data in all seven assessments; Austria, Chile, Estonia, Israel, Luxembourg, the Netherlands, the Slovak Republic, Slovenia, Spain, Turkey, the United Kingdom and the United States are not included in this average. Source: OECD, PISA 2018 Database, Table I.B1.10.

An obvious limitation of the existing knowledge base is that it focuses on particular places and programs, and attempts to extrapolate findings from those studies to different places and programs assumes a generalizability beyond the strict empirical boundaries within which such knowledge was gained. In particular, transfering knowledge about teacher professional development across national education systems should be done carefully (Villegas-Reimers, 2003). The varying results observed across different countries in their efforts to improve educational outcomes underscores the limitations of existing knowledge about how to build system capacity. The latest PISA study shows that, among the countries that have participated in the study over the last two decades, there are many different patterns of improvement, as well as many different patterns of decline. In some countries, student performance is today very comparable to what it was two decades ago. In other countries, such performance has declined. In others in has increased. Figure 1.2 shows these various patterns.

These results are helpful to place what is known about strategies to improve systems to teach a broader range of competencies in context. Much of the education research-based knowledge originates in countries, such as Canada and the United States, in which there has been relatively little change in student learning outcomes as assessed in PISA over the last two decades. Arguably, this could help understand why some scholars of the process of educational change in those settings have concluded that reform at scale is more likely to fail than to succeed at changing the basic grammar of schooling (Elmore, 1996; Olson, 2003; Tyack & Cuban, 1997; Tyack & Tobin, 1994).

In other countries which have been well represented in the educational research literature, such as Australia or Finland, the student learning outcomes of students in PISA shows a pattern of consistent decline over the last two decades, albeit from relatively high levels of performance.

In contrast, relatively little educational research has focused on the approaches followed to improve school in countries where students show improvement in PISA scores over these two decades such as Jordan, Macao, Russia, Estonia, Portugal, or countries where students showed improvement during part of that period such as Albania, Chile, Colombia, Germany, Israel, Montenegro, Peru, Poland, Qatar or Romania.

It is therefore necessary to keep in mind that the prevailing conventional wisdom emerging from educational research may be biased by the nature of the educational systems where such research has been conducted in. The world is a much richer laboratory of educational ideas and practices than is covered by existing educational scholarship, much of which focuses in countries where improvement is elusive, while countries where improvement is observed are understudied.

In addition to the limitations of the existing knowledge base stemming from the relatively few countries covered by such literature, transferring ideas about ‘what works’ in system level change, teacher education and professional development across contexts should be done with great care. There are differences across systems in the rules and norms that shape who practices the profession. In some settings, teachers have more limited general knowledge than in others, they vary in professional preparation, the characteristics of the schools in which they work differ, this variation is also a reason to be curious about what works in different settings.

It is especially important to build a knowledge base about how best to support teachers and system level improvement in the developing world because 90% of the world’s children attend schools there. We should not assume that what has proven to work in countries where higher levels of resources are available to support teachers may easily transfer to or be sustainable in contexts where the levels of resources are more limited or where the nature of educational institutions differs. We should be agnostic as to whether the same practices to support teacher professionalism apply in contexts where the extent and nature of the politization of education differ, or about whether changes in requirements for teacher preparation have similar effects in countries where governments have vastly different authority over teacher preparation institutions.

An example from the field of sanitation will illustrate the point that sometimes an approach or a technology that addresses a problem in one setting does not transfer well to a different setting, particularly when resource constrains are critical to the ability to solve the problem at scale. In his efforts to improve sanitation in the developing world, Bill Gates concluded that the toilets and water treatment systems developed and in use in the early industrialized world were poor fits to developing countries because they were resource intensive and generated excessive waste. This caused him to undertake projects to stimulate innovation in the design of next-generation toilets that could operate without sewer systems (Brueck, 2019; D’Agostino, 2018). This approach shows that in order to achieve the same public health results, attempting to transfer a technology developed at a particular time for a particular context, with a given level of resources and institutional capacity, to a different context would be suboptimal to inventing a new technology, designed specifically to address the needs and constrains of those contexts.

Developing countries face different education challenges than early industrialized countries. Because of growing populations and limited institutional capacity in the education sector, often they must attend to expanding access, while also improving quality. This creates different demands and constrains than improving quality in a context in which the population of school children is stable or declining. An expanding system may have to appoint teachers with relatively limited knowledge and skills, simply because the demand for teachers exceeds the availability of qualified candidates. The challenges faced by teachers are very different in a context where parents have comparable levels of education to those of teachers, than in contexts where many parents have very low levels of education. Levels of financing are also likely to influence the conditions of schools, as well as the nature of the infrastructure and resources in schools. General conditions of development in the communities in which schools work also create demands on teachers, they influence how easy it is for them to access school, but also what challenges the students that they teach experience. In settings of great needs there are demands placed on teachers to fill roles which go beyond the instructional role, in service of their students, such as participating in vaccination campaigns or in nutritional programs. The level of institutionalization of education can also influence demands on teachers, for instance, in some settings national politics have more pervasive effects than in others on teacher appointments and translate into demands for teachers to participate in political activities.

As developing countries advance reforms to develop twenty-first century competencies, of deeper learning and educating the whole child, even as they still address twentieth century challenges of access and basic literacies, we need to better understand what it is like to implement ambitious curriculum reforms in a context with over-crowded classrooms, poorly paid teachers and underfunded systems, with weak institutional capacity, or in systems where there is political or economic instability, or where the education system has been captured by vested interests and where patronage and corruption are rampant. This comparative analysis might help us discern which features of the implementation of system level reforms are sufficiently robust to ‘work’ across contexts, approaches at least worth trying, and which approaches might necessitate more careful sequencing, in which certain pre-conditions are first established before some components of reform can be pursued.

In order to determine what might be the best way to implement twenty-first century education reforms across these various contexts, we need to study reforms across vastly different contexts, not just on a limited set of similar countries. This book is an effort in that direction. We examine system level reforms across a range of different national education systems. The main thrust of the book is descriptive, providing a careful account of the details of implementation in each of the six countries studied. We also characterize the system level reforms in terms of whether they address cultural, psychological, professional, institutional and political dimensions of the change process. Finally, the concluding chapter draws some lessons about the implementation of reforms based on a cross-case analysis of the six cases.

## Methods of This Study

The six cases of reform examined in this book were initiated as part of a graduate course in education policy analysis I teach at the Harvard Graduate School of Education. The authors are all experienced educators with professional experience as teachers, coaches, providers of teacher professional development, education leaders, government staff, education consultants for governments and international organizations. Collectively they have worked in Afghanistan, Australia, Bhutan, Burkina Faso, Canada, Central African Republic, Colombia, Cote D’Ivoire, Denmark, Djibouti, France, Guinea, India, Israel, Latvia, Malaysia, Maldives, Mali, Mexico, Niger, Pakistan, Panama, Rwanda, Syria, Switzerland, Tanzania, Thailand, Turkey, UAE, Uganda, United Kingdom, United States, and Zimbabwe.

To conduct these studies, the authors accessed published documents, statistical information, results from comparative studies, and interviewed education officials and researchers in the countries they were studying. Some of the teams also travelled to the countries they were studying for several weeks, after producing a first draft of the study, to collect additional data. Each chapter was then discussed by the entire team involved in studying the six cases, leading to several rounds of revisions to achieve coherence and comparability across chapters.

Upon completion of the course, the findings were presented to various audiences familiar with the contexts studied, including at a conference on global education at which senior leaders of practice discussed the reports and provided feedback. Subsequently each research team revised their chapter to achieve greater integration and coherence within the structure of this book. A final set of revisions followed helpful suggestions from the three anonymous reviewers engaged by Springer to peer review the manuscript.

The studies were informed by the content of the course, which reviewed literature on twenty-first century skills, deeper learning, system level change, curriculum and teacher education policy reform. The course readings included also some of the publications of the Global Education Innovation Initiative, a research and practice consortium I lead with the goal of supporting the transformation of public education systems towards greater relevance. The following guiding questions, which I developed to guide two previous studies of the Global Education Innovation Initiative, were used to frame the case studies:

1. What was the reform about, what time frame was covered by the reform?
2. What sources of evidence were used to conduct the study?
3. What Context preceded and gave rise to the reform? Was this reform part of the agenda of a new government? A response to an economic crisis? What were the educational antecedents of this reform? What were the factors which gave impetus to a reform agenda? Where there international influences of any sort? Did international evidence or ideas influence the context?
4. Description of the reform: what were the intended goals, what were the key components of this reform (change in law, budget, curriculum, assessment, etc.), what was the underlying theory of change of the reform? Who participated in the design of the reform and in implementation?
5. In what way did the educational goals of the country’s reform relate to the idea of twenty-first century skills or breadth of skills or cognitive and socio-emotional development? Which specific outcomes and skills were emphasized in the reform?
6. Which specific components of the reform are directly related with the development of twenty-first century skills in students? How are they implemented? Description of specific programs that develop twenty-first century skills. (Curriculum, assessment, school autonomy, partnerships, specific programs in schools such as project based learning or specific programs of teacher professional development)
7. What were the various stages of implementation of the reform? Who participated? How are governments (federal/local) coordinating with other stakeholders?
8. What is known about the politics of the reform? Which factors supported implementation? Which impeded it?
9. What do we know about the results of the reform achieved so far? Have they been evaluated? What are the challenges?

## Five Perspectives on Educational Change

Implementing educational policies depends greatly on communication and collaboration across a large number of stakeholders in the education system. They include those who initiate change, as well as those who implement change, they involve people at different levels of the system: in classrooms, school districts, various administrative levels, and senior leadership roles. Each of these actors makes sense of the change process through one or multiple frameworks, whether they are aware of it or not. I believe that deliberate attention to these frameworks, to what each of them reveals and to what they conceal, can help each actor better discern how to implement educational change, as well as better understand other stakeholders involved in the change process, and communicate with them.

To characterize these frameworks, I have developed a conceptual model that analyzes education reforms through five complementary lenses: **cultural, psychological, professional, institutional, or political**. (Reimers, 2020c). A cultural frame highlights the correspondence between societal demands and values and the proposed educational change; a psychological frame focuses on the use of the science of learning and teaching in the design of the change process; a professional frame reflects the creation of norms and processes designed to align professional practice with expertise; an institutional frame focuses on the process of educational change as the result of a system of interdependent processes and a political frame focuses on the role played by various interest groups in advancing or impeding educational change.

These frames serve as analytic tools to conceptualize change. In practice, any reform effort may reflect the reliance on strategies that are best understood through more than one frame. I applied those frames in a comparative study of curriculum reforms in Brazil, Finland, Japan, Mexico, Peru, Poland, Portugal, and Russia, and found that all of them reflected the use of strategies that illustrated more than one frame, but that none of them reflected strategies illustrating a comprehensive use of all five of them (Reimers, 2020b). Furthermore, I also found that institutional and political perspectives were more commonly used by reformers than cultural or psychological perspectives. Increasingly, professional perspectives are also in use as the importance of teacher expertise is recognized as critical to implementing ambitious reform efforts.

Another study of the Global Education Innovation Initiative, a compilation of reflections from system level leaders who had attempted ambitious education reform designed to make visible their theories of action, showed that institutional and political perspectives were dominant in their accounts (Reimers, 2019).

One of the contributors to that compilation, a former secretary of education of the city of Rio de Janeiro, in Brazil, summarizes her approach in a way that illustrates reliance on a professional and institutional perspective:

Thus, two efforts had to be undertaken simultaneously: starting to build a culture of excellence and of high expectations for every student, and implementing affirmative action to ensure that the most challenged schools received additional support. The approach taken was inspired by Michael Fullan’s recommendation of a systemic transformation when reforming the education in a city, so as to avoid fragmentation or the improvement of just some areas in a complex setting [….] With this approach in mind, my team and I developed a program based on the following principles:

- Schools should collaborate with one another, in an ecosystem of learning;
- Teachers should participate in the design of the curriculum, in the preparation of the textbooks to be used to support their practice, in the elaboration of the digital classes that were to be inserted in a platform to make teaching and learning more interesting, in the assessments, and in the elaboration of a remedial education course for the students who were not learning;
- We would open the system for experimentation, trying to find scalable good practices;
- Formative assessment would be incentivized and a unified test would be implemented in all schools every 2 months, with questions prepared collectively by teachers from different schools, to ensure that students were progressing as expected;
- Good teaching would be made visible, not only to the system, but to the whole city; and
- Equity and inclusion would be our most valued principles, alongside excellence. (Costin, 2019, 39–40).

Another contributor to that book, a former minister of education of Colombia, summarizes her approach through an institutional frame, and maintaining strategic focus as a way to prevent the capture of the change effort by various political interests:

we developed a program that increased enrollment at all educational levels, we developed a quality improvement system, and we modernized the management of the sector.” (Velez, 2019, 51). “We had the strong conviction that it was crucial to undertake an institutional change that allowed the sector to obtain the results that were set in the plan and to make the change sustainable. An important chapter of our plan was focused on aligning institutions to their objectives. The parallel structures (that may be necessary to start a project) are ephemeral and do not guarantee long-term actions, which is the work needed in education. (Ibid, 54).

Several of these system level leaders use political frameworks to analyze their efforts, as is the case with a former Minister of Education of Mexico:

Unlike economic or financial markets, where you are always looking to increase and save your own resources, the opposite should be done in politics: you must spend your political capital at the beginning of your term, when this capital is possibly at its highest, to make difficult –but important– decisions, even if they are unpopular. The explanation is simple: your political power will vanish quickly, your incumbents will be cruel and brutal, and political circumstances will likely change. (Granados, 2019, 85).

Recognizing that education reforms can be used to advance personal political aims he advises future ministers to forget their political ambitions and stop campaigning.

Also relying on a political framework, a former Minister of Education of Peru, begins his reflection in this way:

If there is no political alignment behind education, you will have to fight to position education so that these constituencies understand its importance. Education is about giving the right quality service to all children and youth, and as such, it is a long-run endeavor that requires the full political commitment of the executive, of parliament, and of society in general. (Saavedra, 2019, 108).

## Content of This Book

This book examines six system level reforms which all intended to improve instruction by expanding the depth and breadth of the curriculum. All of them addressed teacher professional preparation as part of the strategy of implementing the new curriculum, although they did it in different ways and with different results. In addition, some of them also enacted institutional reforms as a way to support the implementation of more ambitious curricula and to support increased teacher capacity, as with teacher professional preparation, there were important differences in the details of those reforms.

In Ontario, the provincial government developed and implemented a reform designed to improve the quality of instruction depending largely on providing teachers opportunities to co-construct the improvement process, and in promoting accountability and coherence across various levels of the system through the use of information. This reform took place in a context in which a new political administration attempted to create more collaborative approaches with teachers and teacher unions, following a contentious period. The focus of the reform was on the improvement of literacy, numeracy, and high school graduation rates. The main drivers of the reform were capacity building and accountability.

The reform adopted a learning orientation, of refining and adapting implementation as a result of the observed results of the actions undertaken. While these were not original goals of the reform, partway through the reform process, the development of twenty-first century skills became a goal in some provinces, as a result of a growing movement of deep learning, however there was no explicit emphasis on twenty-first century skills in policy, curriculum, or assessment. This reform illustrates the use primarily of political, professional and institutional perspectives in that it sought to align key stakeholders and to construct collaborative relationships between teachers and administrators, it sought to empower teachers as professionals and build on their professionalism and it sought to develop institutional capacity and coherence through the use of information. There is also some evidence of reliance on a psychological perspective.

In Singapore, the Government redesigned teacher initial preparation in line with a capacious vision for education which emphasized twenty-first century skills. The reform emphasized the holistic development of the teacher during their initial preparation, so they could in turn educate whole students. Because teachers are initially prepared in a single institution, the National Institute of Education, Nanyang Technological University, Singapore (NIE NTU, Singapore) it was possible to implement this new teacher preparation curriculum with great fidelity and coherence. The emphasis of this reform reflects a professional perspective, enhanced by an institutional perspective as there were other changes ongoing that supported the reform of teacher education, such as a curriculum reform. The chapter highlights how Singapore leveraged emerging ideas from international education organizations and think tanks on education for the twenty-first century to shape its own education strategy.

In Mexico, as part of a comprehensive set of structural reforms that sought to increase the competitiveness of Mexico’s economy and to address inequality, the government advanced a comprehensive education reform which included a clear focus on a broad set of competencies. A key element of that reform included taking control over teacher appointments and careers away from the teacher union, which generated predictable opposition from the teacher union. The reform was approached primarily through a political and an institutional perspective. The sequence of reform, and the short tenure of the administration, limited effective use of a cultural, psychological or professional perspective, furthering the political opposition to the reform. While the reform included also a program of teacher evaluation and preparation, and repurposed an institute of educational evaluation to undertake the core functions of teacher support, these components of the reform strategy came too late in the tenure of the government advancing the reform to be fully or effectively implemented.

In Pakistan, the Punjab Education Reform was, essentially, an institutional capacity building strategy aimed at overhauling the delivery and monitoring systems in the province. The reform did not address breadth of skills, or curriculum, but rather increased the capacity of schools to deliver the existing curriculum strengthening capacity and accountability for access, literacy and numeracy. Following the approach to whole system reform used in the United Kingdom during the Tony Blair administration, the Punjab Education Reform established a delivery unit to maintain focus on the implementation of a limited set of reforms. The reform built management capacity as a way to strengthen the delivery chain to implement the reform goals, it also built teacher capacity, reflecting elements of a professional perspective. A decade into implementation of this reform, it was still not explicitly addressing twenty-first century skills.

In Kenya, the government implemented a competency-based national curriculum reform in 2017 intending to develop a broader range of competencies in line with supporting Kenya’s greater competitiveness. It piloted the reform in 470 schools, for broader roll out subsequently. They also used a train-the-trainer (ToT) model for teachers’ training and preparation. Part of the reform included a structural change to education, from 8 years of primary and 4 years of secondary education to 6 years of primary, 3 of middle, and 3 of tertiary education, to provide greater focus on technical and vocational education. The strategy adopted by the reform reflects a cultural and a political perspective. While the reform aimed at building teacher capacity, those efforts have been insufficient to equip teachers to teach the more ambitious competency-based curriculum.

In Zimbabwe, the government developed a new set of standards and curriculum frameworks with the aim of better equipping students for the evolving needs of the twenty-first century. To support the implementation of the new curriculum, the Ministry distributed school packages which included the curriculum framework, the syllabi and assessment resources. Challenges with the delivery of those packages, however, have limited the broad dissemination of the curriculum among teachers. The reform contemplates professional development for teachers, which is currently being implemented, as well as preparation and distribution of instructional materials and assessment, which have not yet taken place. The reform illustrates the use of an institutional and a professional perspective, roles for teachers and school administrators were redesigned to align them to the new curriculum goals.

To sum up, while an ambitious set of education goals guides reforms in Singapore, Mexico, Kenya and Zimbabwe, only in Singapore a relatively small system in which there is great institutional coherence and significant educational investment in education, is there evidence that teacher preparation programs are aligned with those ambitions. In Mexico, the sequencing of the reform prioritized the establishment of professional norms for teacher appointments and promotions. The contention generated by those changes, and a political transition, aborted the full implementation of the planned supports to build teacher capacity. In Kenya and Zimbabwe, the ambitious of the curriculum reforms greatly exceed the supports put in place to build institutional capacity.

If implementation of twenty-first century education was elusive in these four countries were policy declared the intent to pursue it, things were not much different in the two jurisdictions which chose first to build system capacity focused on the basic literacies: Ontario and Punjab. After a long period of building capacity, those systems had improved in the goals they had set out to improve, but they had not transitioned to pursue the breadth of skills advocated by the various organizations discussed in this chapter.

Which leaves us in a difficult place, concluding that twenty-first century education remains an elusive goal, one embraced rhetorically by reforms of education systems at various stages of implementation, but not yet reflected in implementation strategies which could possibly match those ambitions. In the chapters that follow we examine the details of the implementation of those reforms.
